# Spatiotemporally distinct roles of cyclooxygenase-1 and cyclooxygenase-2 at fetomaternal interface in mice

**DOI:** 10.1172/jci.insight.181865

**Published:** 2024-08-27

**Authors:** Shizu Aikawa, Mitsunori Matsuo, Shun Akaeda, Yukihiko Sugimoto, Makoto Arita, Yosuke Isobe, Yuki Sugiura, Shu Taira, Rae Maeda, Ryoko Shimizu-Hirota, Norihiko Takeda, Daiki Hiratsuka, Xueting He, Chihiro Ishizawa, Rei Iida, Yamato Fukui, Takehiro Hiraoka, Miyuki Harada, Osamu Wada-Hiraike, Yutaka Osuga, Yasushi Hirota

**Affiliations:** 1 Department of Obstetrics and Gynecology, Graduate School of Medicine, The University of Tokyo, Tokyo, Japan.; 2 Department of Pharmaceutical Biochemistry, Graduate School of Pharmaceutical Sciences, Kumamoto University, Kumamoto, Japan.; 3 Division of Physiological Chemistry and Metabolism, Graduate School of Pharmaceutical Sciences, Keio University, Tokyo, Japan.; 4 Laboratory for Metabolomics, RIKEN Center for Integrative Medical Sciences, Kanagawa, Japan.; 5 Cellular and Molecular Epigenetics Laboratory, Graduate School of Medical Life Science, Yokohama City University, Kanagawa, Japan.; 6 Division of Multiomics Platform, Center for Cancer Immunotherapy and Immunobiology, Graduate School of Medicine, Kyoto University, Kyoto, Japan.; 7 Faculty of Food and Agricultural Sciences, Fukushima University, Fukushima, Japan.; 8 Department of Internal Medicine, Center for Preventive Medicine, Keio University School of Medicine, Tokyo, Japan.; 9 Department of Cardiovascular Medicine, Graduate School of Medicine, The University of Tokyo, Tokyo, Japan.

**Keywords:** Endocrinology, Reproductive biology, Eicosanoids, Reproductive biochemistry

## Abstract

Embryo implantation is crucial for ensuring a successful pregnancy outcome and subsequent child health. The intrauterine environment during the peri-implantation period shows drastic changes in gene expression and cellular metabolism in response to hormonal stimuli and reciprocal communication with embryos. Here, we performed spatial transcriptomic analysis to elucidate the mechanisms underlying embryo implantation. Transcriptome data revealed that lipid metabolism pathways, especially arachidonic acid–related (AA-related) ones, were enriched in the embryo-receptive luminal epithelia. Cyclooxygenases (COXs), rate-limiting enzymes involved in prostaglandin production by AA, were spatiotemporally regulated in the vicinity of embryos during implantation, but the role of each COX isozyme in the uterus for successful pregnancy was unclear. We established uterine-specific *COX2*-knockout (uKO) and *COX1*/uterine *COX2*-double-KO (*COX1*/*COX2*-DKO) mice. *COX2* uKO caused deferred implantation with failed trophoblast invasion, resulting in subfertility with reduced pregnancy rates and litter sizes. *COX1/COX2* DKO induced complete infertility, owing to abrogated embryo attachment. These results demonstrate that both isozymes have distinct roles during embryo implantation. Spatial transcriptome and lipidome analyses revealed unique profiles of prostaglandin synthesis by each COX isozyme and spatiotemporal expression patterns of downstream receptors throughout the endometrium. Our findings reveal previously unappreciated roles of COXs at the fetomaternal interface to establish early pregnancy.

## Abstract 2

Cox-1 and Cox-2 at feto-maternal interface differentially contribute to embryo spacing and invasion to establish early pregnancy by synthesizing unique PG species.

## Introduction

**Figure 1:**
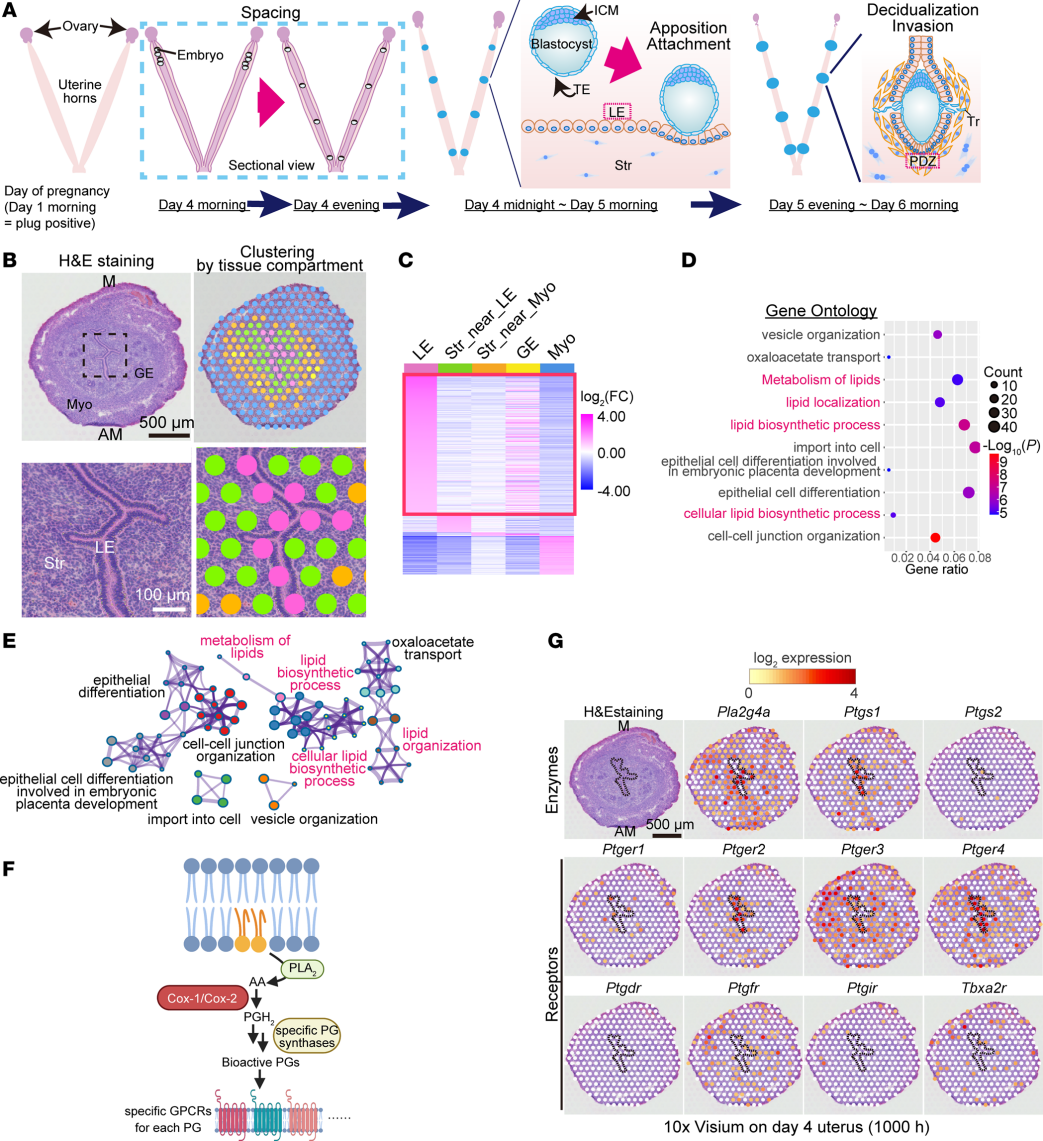
Spatial transcriptome revealing that lipid metabolism pathways are enriched in LE before embryo attachment. (**A**) Schematic diagram of the process of embryo implantation in mice. ICM, inner cell mass; TE, trophectoderm; Tr, trophoblast; LE, luminal epithelia; Str, stroma; PDZ, primary decidual zone. (**B** and **C**) Spatial transcriptome using 10× Visium revealed LE-specific genes on day 4. In **B**, the area demarcated by a dashed line in the upper panels were magnified further in the lower panels. M, mesometrial pole; AM, anti-mesometrial pole; GE, glandular epithelia; Myo, myometria. Scale bars: 500 μm (upper in **B**) and 100 μm (lower in **B**). See also Supplemental Table 1. (**D** and **E**) Gene Ontology (GO) analyses using Metascape showed that LE-specific genes were enriched in lipid metabolism–related pathways. See also Supplemental Table 2. (**F**) Schematic diagram of PG synthesis from arachidonic acid (AA) and downstream GPCRs. (**G**) Spatial transcriptome of day 4 uteri showing unique expression patterns of PG synthesis–related enzymes (top) and receptors (middle and bottom). The area of LE is encircled by a dashed line. Scale bar: 500 μm. Day 4 (1000 hours) indicates 10 am on day 4 of pregnancy.

Successful pregnancy requires appropriate conditions in the endometrium and healthy embryos (1, 2). Embryo implantation is a key process in which the embryos and uterine tissues dynamically interact (Figure 1A). In mice, the blastocyst arrives in the uterus in the early morning of day 4 of pregnancy (day 1 = plug-positive). Blastocysts hatch from their surrounding zona pellucida and have proper spacing between each embryo by the evening of day 4 (3). Embryos then interdigitate with the surface of the uterine luminal cells; this process is termed apposition. Once the blastocyst strongly attaches to the uterine epithelium on day 4 at midnight, the surrounding stromal cells initiate differentiation into decidual cells (decidualization). By the evening of day 5, trophoblast cells would have invaded the decidua by removing the surrounding luminal epithelial cell layer, eventually contributing to placenta formation. Any defects in the implantation and decidualization processes will have ripple effects on pregnancy outcomes; therefore, the interaction between the blastocyst and endometrium during early pregnancy must be tightly regulated (1). While accumulating studies using genetically engineered mice have revealed important genes that regulate embryo implantation (1, 2), it remains elusive how the expression of uterine genes changes spatiotemporally during the peri-implantation period.

It is recognized that nonsteroidal antiinflammatory drugs (NSAIDs) impair early pregnancy events, including implantation and decidualization; hence, the intake of NSAIDs is prohibited in pregnant women (4). NSAIDs inhibit the enzymatic activities of cyclooxygenase 1 (COX1) and COX2, the rate-limiting enzymes in the synthesis of prostaglandins (PGs) from arachidonic acid (AA). PGs are bioactive lipids that exert their effects by activating specific cell membrane–spanning receptors (4–6). While COX1 and COX2 share structural features, with approximately 60% sequence homology, each enzyme shows a unique localization at both the tissue and subcellular levels (7). COX1 is considered the “housekeeping” COX, as it is expressed constitutively in most tissues, whereas COX2 expression is inducible by particular stimuli, such as inflammation and intracellular Ca^2+^ mobilization (5, 7). In cells, COX1 tends to localize to the endoplasmic reticulum, whereas COX2 preferably localizes to the perinuclear membrane. These differences in intracellular localization are considered to reflect the unique biological role of each isozyme. Indeed, in vitro studies have demonstrated that COX1 and COX2 have different substrate preferences, resulting in the synthesis of unique PG species (7). However, the functional differences between the 2 COXs in vivo have not been well clarified. In mouse uteri, cytosolic phospholipase A_2_α (cPLA_2_α) and COX1 are highly expressed in the luminal epithelium on day 4 in the morning. After embryo attachment, COX1 disappears, but COX2 colocalizes with cPLA_2_α at epithelial and stromal cells surrounding the embryos (8). These expression patterns suggest that COX1 and COX2 have differential roles in establishing early pregnancy. However, the differential functions of COXs in the uterus remain unclear (6) for the following reasons. First, in *COX1*-knockout (*COX1*-KO) uteri, no severe defects in early pregnancy were observed because COX2 compensated for the lost COX1 function (9, 10). Second, females lacking COX2 systemically are completely infertile due to defects in ovulation and fertilization (11); therefore, the uterine-specific roles of COX2 are obscure.

In this study, we conducted spatial transcriptomic analysis on mouse uteri and found that gene expression in lipid metabolism pathways, especially AA-related ones, are enriched at receptive luminal cells and primary decidual zones (PDZs), which directly contact to blastocysts. In addition, the spatiotemporal expression of COX1/COX2 and PG receptors was dramatically altered during the implantation processes. Lipidome analyses using liquid chromatography with tandem mass spectrometry (LC-MS/MS) and imaging MS also revealed that COX1 and COX2 uniquely play roles at different time points. To elucidate the differential roles of COX1 and COX2, we established uterine-specific *COX2*-KO (uKO) and *COX1*-KO/*COX2*-uKO double-KO (*COX1*/*COX2*-DKO) mice to investigate the functions of these 2 enzymes during pregnancy.

## Results

### Spatial transcriptome reveals AA/PG-related pathways in the fetomaternal interface during the embryo implantation period.

On the morning of day 4, blastocysts and endometria begin to physically communicate to establish embryo implantation (Figure 1A) (1). Luminal epithelia (LE) have direct contacts with embryos until they are eliminated after embryo attachment (Figure 1A) (12, 13), indicating critical roles for LE in embryo spacing and attachment.

To elucidate the landscape of signaling networks governing the functions of LE on day 4, we performed spatial transcriptome analysis using 10× Visium (Figure 1, B and C). Gene clustering according to the tissue compartment on the morning of day 4 revealed uniquely expressed genes in LE compared with that in other cell types (Figure 1C and Supplemental Table 1; supplemental material available online with this article; https://doi.org/10.1172/jci.insight.181865DS1). We validated our 10× Visium data by immunostaining of cytokeratin 8 (CK-8; an epithelial marker), Ki67 (a proliferation marker; stromal cells should be stained on day 4; ref. 14), and Foxa2 (a glandular epithelium marker) (Supplemental Figure 1). Gene Ontology (GO) analyses showed that LE-unique genes were enriched in lipid metabolism–related pathways (Figure 1, D and E, and Supplemental Table 2). Notably, AA-related enzymes (e.g., *Pla2g4a*, *Pla2g10*, *Lpcat3*, and *Acsl4*) and receptors (*Ptger2* and *Ptger4*) of PGs were detected (Supplemental Tables 1 and 2). Wang et al. (15) recently reported concentrated lipid metabolism in LE via single-cell RNA-sequencing (scRNA-seq) analyses. We reanalyzed their scRNA-seq data from mouse endometria on day 4 (3.5 day post coitus [dpc] in the original paper) and found that AA-related enzymes were highly expressed in LE (Supplemental Figure 2), supporting our 10× Visium data.

Transcriptome analyses enabled observation of the spatial expression of PG synthesis–related enzymes and downstream receptors on the morning of day 4 (Figure 1, F and G). In the mouse uterus, cPLA_2_α (encoded by *Pla2g4a*) is responsible for AA supply for PG synthesis (Figure 1F). Female mice lacking cPLA_2_α show deferred embryo implantation and crowded embryo spacing, resulting in shared placentae and reduced litter sizes (8). In agreement with this, our spatial transcriptome analysis showed prominent expression of *Pla2g4a* (cPLA_2_α) in LE with *Ptgs1* (COX1), indicating crucial roles for cPLA_2_α and COX1 in these epithelia (Figure 1G). In contrast with COX1, the COX2 gene (*Ptgs2*) was poorly expressed in this milieu (Figure 1G). Each PG species produced by COXs triggers unique biological actions by binding to specific G protein–coupled receptors (GPCRs), although it remains unclear which PG receptor(s) is crucial for embryo implantation (4). The transcriptome data revealed that PGE_2_ receptors *Ptger2*, *Ptger*3, and *Ptger4* were highly expressed on the morning of day 4, while *Ptger1* showed a faint expression (Figure 1G). Notably, *Ptger2* and *Ptger4* were expressed on the luminal epithelium, where COX1 is also expressed (Figure 1G), suggesting possible roles of the COX1/PGE_2_ axis in implantation events in day 4 uteri. In contrast with the high expression of PGE_2_ receptors, other PG receptors, *Ptgdr* (a PGD_2_ receptor) and *Ptgir* (a PGI_2_ receptor), were faintly expressed (Figure 1G). PGD_2_ and PGI_2_ have another receptor, *Ptgdr2* and *Ppard*, respectively (16, 17). Similar to *Ptgdr*, *Ptgdr2* also showed poor expression in day 4 uteri (Supplemental Figure 3). *Ppard* encodes the nuclear receptor PPARδ (17). We found stromal expression of *Ppard* (Supplemental Figure 3). This observation agrees with that of a previous study demonstrating that PGI_2_ works in early pregnant uteri via PPARδ rather than GPCR (18); it is, however, unclear whether the PGI_2_/PPARδ axis works physiologically (19). The 2 remaining PG receptors, *Ptgfr* (a PGF_2α_ receptor) and *Tbxa2r* (a thromboxane A_2_ [TxA_2_] receptor), were majorly found in the myometrial layer.

After embryo attachment, stromal cells undergo differentiation called decidualization (1) (Figure 1A). The decidua in the close vicinity of the attached blastocysts is the PDZ, which exhibits epithelial cell–like features with tight junctions (20, 21). The PDZ is considered important to protect embryos from maternal immune cells and pathogens instead of eliminated LE after embryo attachment (Figure 1A) (12, 13). In uteri with abnormal PDZ formation, embryo growth is compromised, with defective embryo invasion (21, 22), indicating the importance of the PDZ during the embryo invasion phase.

**Figure 2:**
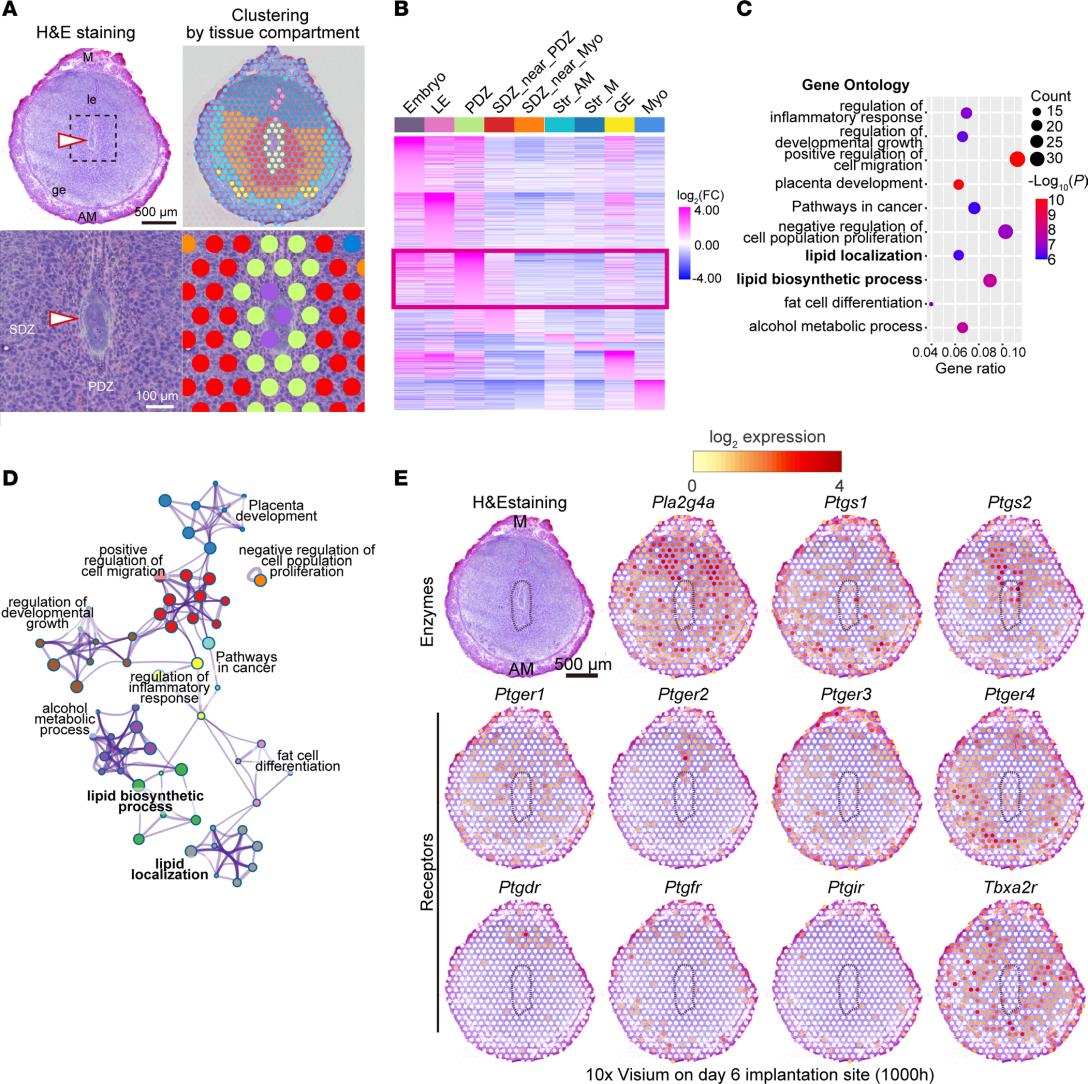
Enriched lipid-related pathways in PDZ during the embryo invasion phase. (**A** and **B**) Spatial transcriptome using 10× Visium revealed PDZ-specific genes on day 6. In **A**, the area demarcated by a dashed line in the upper panels was magnified further in the lower panels. M, mesometrial pole; AM, anti-mesometrial pole; LE, luminal epithelia; GE, glandular epithelia; Str, stroma; PDZ, primary decidual zone; SDZ, secondary decidual zone; Myo, myometria. Arrowheads indicate embryos. Scale bars: 500 μm (upper in **A**) and 100 μm (lower in **A**). See also Supplemental Table 3. (**C** and **D**) Gene Ontology (GO) analyses using Metascape identified PDZ-specific genes enriched in lipid metabolism–related pathways. See also Supplemental Table 4. (**E**) Spatial transcriptome of day 6 implantation sites, showing unique expression patterns of PG synthetic enzymes (top) and receptors (middle and bottom). PDZ is encircled by a dashed line. Scale bar: 500 μm. Day 6 (1000 hours) indicates 10 am on day 6 of pregnancy.

These results prompted us to investigate the gene network underlying PDZ functions in day 6 pregnant uteri (Figure 2). Our 10× Visium analyses revealed uniquely expressed genes in the PDZ (Figure 2, A and B, and Supplemental Table 3). The cell clustering was validated by immunostaining of *Cdh1* (an epithelial marker) and *Ptgs2* (a PDZ marker) as well as in situ hybridization of *Bmp2* (a secondary decidual zone [SDZ] marker) (Supplemental Figure 4). Gene ontologies for these PDZ-specific genes showed high correlation with lipid metabolism–related pathways (Figure 2, C and D, and Supplemental Table 4). Notably, *Ptgs2*, which encodes COX2, was involved in most of the enriched GO pathways (Supplemental Table 4), indicating a critical role for COX2 in the PDZ. In agreement with our notion, *Ptgs2* was highly accumulated in the endometrium in the vicinity of the attached embryo (Figure 2E), which was in contrast with the poor expression of this gene in day 4 uteri (Figure 1G). The possible contribution of COX2 was further assessed by spatial transcriptome analysis of each PG receptor (Figure 2E). Four PGE_2_ receptors as well as the PGD_2_ receptor were expressed in the vicinity of COX2 (Figure 2E). Notably, *Ptger2* and *Ptgdr* were localized in the epithelium and stroma on the mesometrial side, whereas *Ptger4* was evident in the SDZ and PDZ (Figure 2E). We detected faint expression of *Ptgfr*, *Ptgir* (Figure 2E), and *Ptgdr2* (Supplemental Figure 5). In agreement with a previous report (18), *Ppard* was highly expressed throughout the decidua (Supplemental Figure 5). *Tbxa2r* showed spotty signals throughout the endometrium, but not in the PDZ (Figure 2E), indicating that it is expressed in endothelial cells, as the PDZ is avascular (21). Intriguingly, PGE_2_ receptors were widely distributed in uterine tissues, including the SDZ, whereas the PGD_2_ receptor was localized strictly near the embryo attachment site, where COX2 was also expressed (Figure 2E). This indicated that PGE_2_, but not PGD_2_, can be distributed throughout the endometrium.

### COX1 and COX2 differentially contribute to pregnancy outcomes.

To clarify the uterine roles of COX1 and COX2 during early pregnancy, we established *COX2*-uKO mice by crossing *Ptgs2^fl/fl^* mice with *Pgr*^Cre/+^ mice (Supplemental Figure 6), which do not exhibit ovarian defects, unlike systemic *COX2*-KO mice (11). We also established *COX1*/*COX2*-DKO mice by mating *Ptgs1^–/–^* mice with *Ptgs2^fl/fl^* *Pgr^Cre/+^* ones to assess COX1 functions, while avoiding compensation by increased expression of COX2 in *COX1* single KO (9).

**Figure 3:**
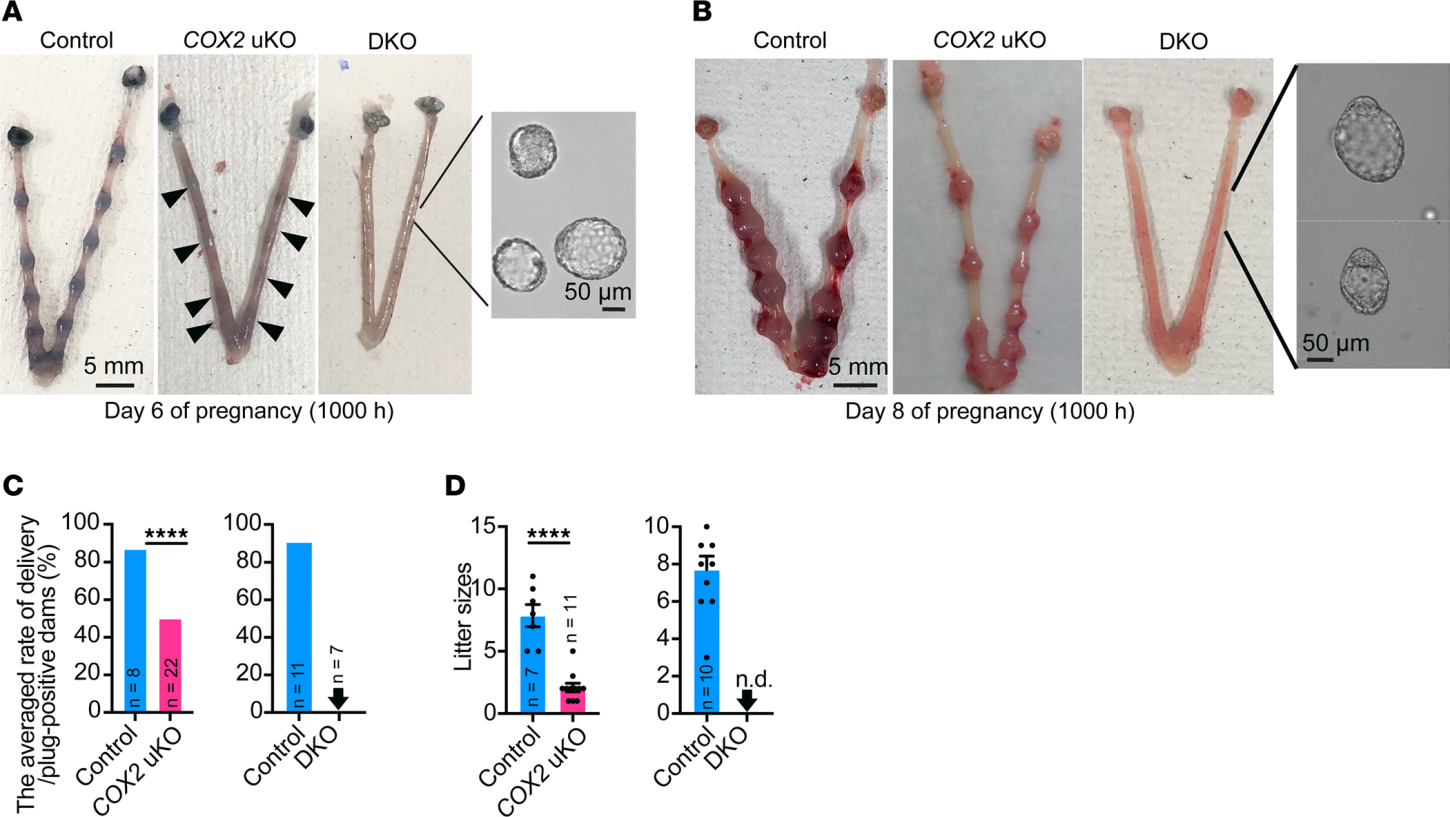
Defective pregnancy phenotypes in *COX2*-uKO and *COX1*/*COX2*-DKO mice. (**A** and **B**) Representative photographs of uteri on day 6 (**A**) and day 8 (**B**). In *COX1*/*COX2*-DKO uteri, unattached blastocysts were flushed from the horns. Scale bars: 5 mm (uteri) and 50 μm (blastocysts). *n* = 3 per genotype. Day 6 (1000 hours) and day 8 (1000 hours) indicate 10 am on each day of pregnancy. (**C** and **D**) Pregnancy rates (**C**) and litter sizes (**D**) indicating subfertility and complete infertility in *COX2*-uKO and DKO mice, respectively. Arrowheads indicate zero value. n.d., not detected. Data are mean ± SEM. ****P < 0.0001 by Student’s *t* test. The number of samples is demonstrated on each bar.

We first explored the physiological impact of COX1, which is highly expressed in luminal cells on the morning of day 4 (Figure 1G). Normally, after embryo attachment, embryo implantation sites can be visualized by an intravenous injection of blue dye due to increased vascular permeability in the endometrium in the vicinity of the attached embryos (23). However, *COX1*/*COX2*-DKO uteri never showed any implantation sites, with unattached blastocysts inside on day 6 of pregnancy (Figure 3A). This phenotype persisted even on day 8 of pregnancy (Figure 3B) when decidual cells reach terminal differentiation (24, 25). Eventually, the *COX1*/*COX2*-DKO mice were completely infertile, as they never delivered pups (Figure 3, C and D). This is in sharp contrast with the phenotype of *COX2*-uKO mice; the uteri from *COX2*-uKO mice had implantation sites both on days 6 and 8 of pregnancy, although blue dye reactions and sizes of each implantation site were smaller than those in the control (Figure 3, A and B). In addition, the average delivery rate in *COX2*-uKO females was 50%, with considerably reduced litter sizes, while it was 87.5% in the control (Figure 3, C and D). These results highlight the critical role of COX1 in the establishment of early pregnancy.

We also observed that deletion of both COX1 and COX2 did not affect ovarian functions and uterine receptivity (Supplemental Figure 7). After ovulation and mating, high serum estrogen (E_2_) levels cause epithelial cell proliferation. By day 3, there is obvious stromal cell proliferation due to increased levels of progesterone (P_4_) secreted from the corpora lutea, while epithelial cell proliferation is terminated. This process, termed proliferation-differentiation switching (PDS), is critical for the establishment of an endometrium receptive for implantation-competent blastocysts (14). Abnormal PDS compromises embryo attachment and invasion (12, 26, 27); therefore, we suspected that PDS would be defective in *COX1*/*COX2*-DKO uteri. However, immunostaining for Ki67 revealed no significant defects in PDS in the mutant uteri on the morning of day 4 (Supplemental Figure 7A). In line with this finding, the expression levels of receptive marker genes in the endometria of *COX2*-uKO and *COX1/COX2*-DKO mice were comparable to those in control mice (Supplemental Figure 7B). Normal serum P_4_ levels further supported the notion that PDS occurred normally in both mutants (Supplemental Figure 7C). In summary, our results suggested a critical role of COX1 in uteri before embryo attachment, while both COX1 and COX2 are dispensable for the establishment of uterine receptivity.

### Critical roles of COX1 in uterine PG synthesis and embryo spacing before embryo attachment.

**Figure 4:**
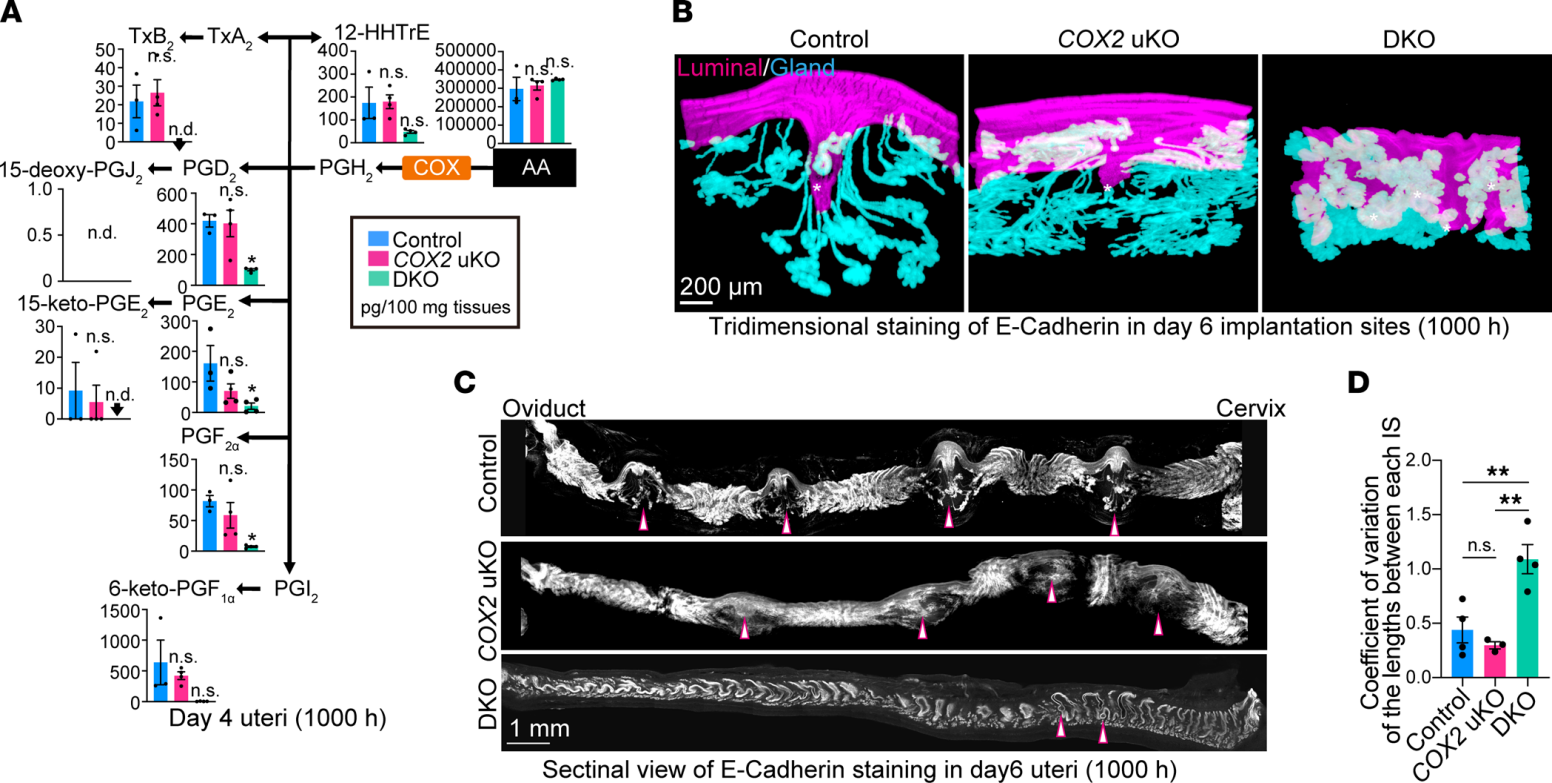
COX1 is crucial for PG synthesis and embryo spacing before embryo attachment. (**A**) Uterine PG concentrations on day 4, as analyzed by LC-MS/MS. *n* = 3 for control and *n* = 4 for *COX2*-uKO and *COX1*/*COX2*-DKO mice. n.d., not detected. Day 4 (1000 hours) indicates 10 am on day 4 of pregnancy. Data are presented as mean ± SEM. *P < 0.05 by 1-way ANOVA followed by Bonferroni’s post hoc test. n.d., not detected; n.s., not significant. (**B** and **C**) 3D images of luminal and glandular epithelia around embryo implantation sites (**B**) and whole uterine horns (**C**) on day 6 of pregnancy. Embryos are indicated by asterisks (**B**) or arrowheads (**C**). Scale bars: 200 μm (**B**) and 1 mm (**C**). At least 3 independent uteri were assessed for each genotype. Day 6 (1000 hours) indicates 10 am on day 6 of pregnancy. (**D**) The average coefficient of variation of the lengths between each implantation site (IS) was calculated from longitudinal scanning images shown in **C**. *n* = 3 for *COX2*-uKO and *n* = 4 for control and DKO. Data are presented as mean ± SEM. **P < 0.01 by 1-way ANOVA followed by Bonferroni’s post hoc test. n.s., not significant.

Next, we tested the enzymatic activities of each COX isozyme in day 4 uteri (Figure 4A). LC-MS/MS analysis found reduced levels of PGD_2_, PGE_2_, PGF_2α_, and TxB_2_ (a stable metabolite of TxA_2_) in *COX1*/*COX2*-DKO uteri (Figure 4A). In contrast, there were no obvious differences in the contents of any PGs between control and *COX2*-uKO mice (Figure 4A). These results were consistent with the fact that COX1, but not COX2, was highly expressed in the luminal epithelium on the morning of day 4 (Figure 1G) and failed embryo attachment was observed in *COX1*/*COX2*-DKO, but not *COX2*-uKO, uteri (Figure 3). According to the expression levels of each specific receptor (Figure 2E), PGE_2_, PGF_2α_, and TxA_2_, but not PGD_2_, might play roles in day 4 uteri.

We then histologically evaluated pregnancy defects in each mutant line (Figure 4, B–D). A 3-dimensional (3D) view of the implantation site provides useful information for detecting anomalies in the implantation process and pregnancy maintenance (28, 29). After embryos attach to the luminal epithelium, the surrounding luminal layer forms crypts at the anti-mesometrial pole, accompanied by elongation of the glands. In uteri with failure of embryo attachment and decidualization, defects in this epithelial shaping have been found (29, 30). Previous studies have shown that embryo spacing occurs by the evening of day 4 (3, 31). Importantly, in *Lpar3*-KO uteri, which show crowded embryo implantation (32), abnormal embryo spacing is already observed on the evening of day 4 and sustained later (3, 31). As embryo implantation sites become evident on day 6 in healthy pregnancy (29), day 6 of pregnancy is the best time to determine whether embryo spacing, attachment, and decidualization are affected. We found poor crypt formation in *COX2*-deleted uteri, in contrast with the deeply evaginated luminal chamber and well-developed glands in control uteri (Figure 4B). *COX1*/*COX2* DKO severely affected the epithelial structure surrounding blastocysts; DKO uteri showed neither crypt shaping nor gland elongation (Figure 4B) and were similar to the uteri before embryo attachment (28, 29). In addition to the epithelial layer abnormalities, blastocysts were located close to each other and to the cervical end in *COX1*/*COX2*-DKO uteri (Figure 4, B–D). Normally, blastocysts are evenly distributed in the uterine cavity during day 4 morning to afternoon (3, 31). Dysregulation of embryo spacing has also been observed in mice deficient in *Lpar3* (encoding a GPCR for lysophosphatidic acid) or *Pla2g4a* (encoding cPLA_2_α), showing a shared placenta and embryo absorption (8, 32). Our observations suggested that COX1-derived PGs are essential for appropriate embryo spacing, while COX2 may play a role after embryo attachment.

### COX2-derived PGD_2_ and PGE_2_ contribute to embryo chamber formation and embryo invasion.

**Figure 5:**
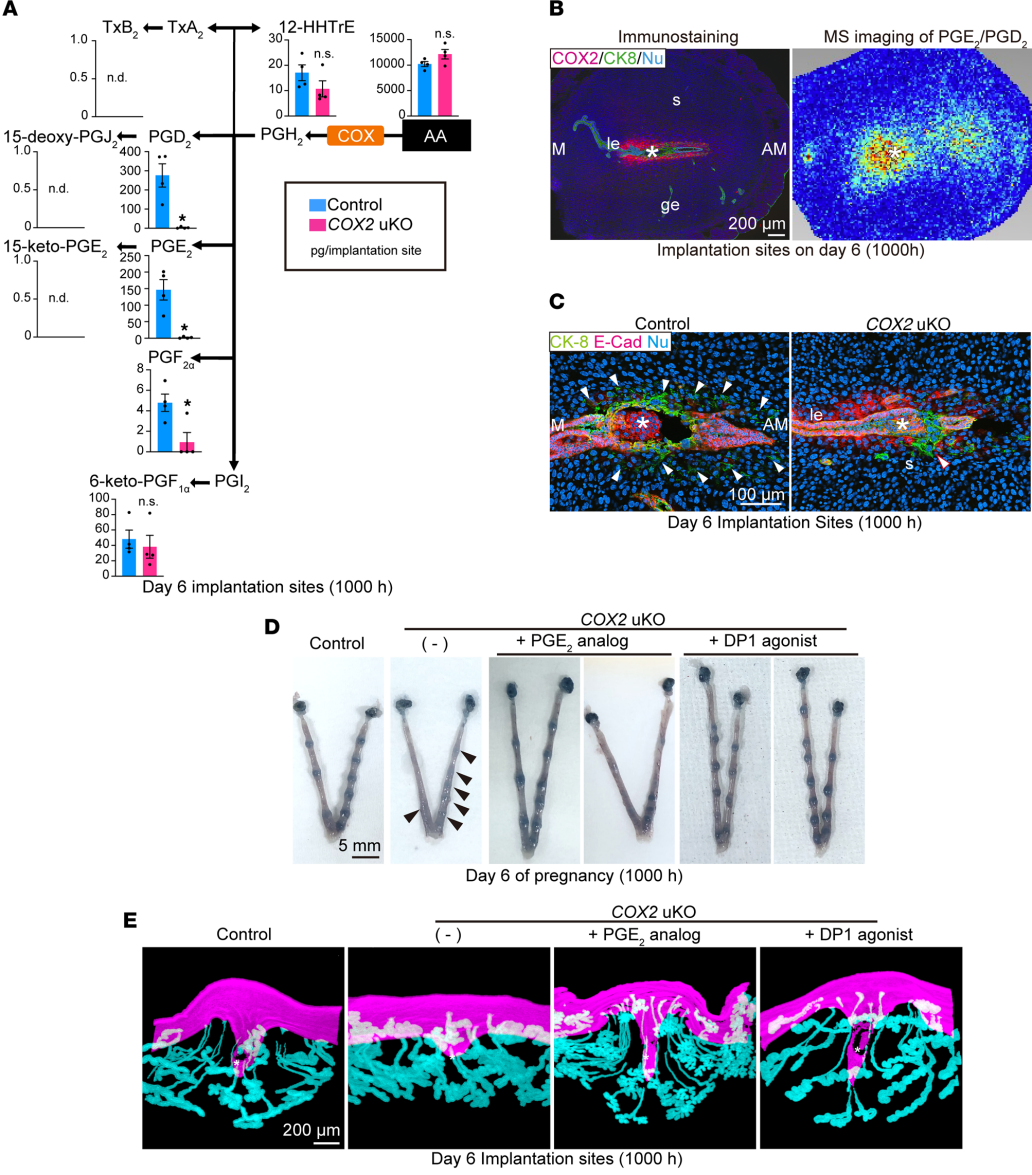
COX2-dependent PG production during the embryo invasion phase. (**A**) PG concentrations in day 6 implantation sites, as analyzed by LC-MS/MS. *n* = 3 per genotype. Data are presented as mean ± SEM. *P < 0.05 by Student’s *t* test. n.d., not detected; n.s., not significant. Day 6 (1000 hours) indicates 10 am on day 6 of pregnancy. (**B**) Immunostaining of COX2 (left) and MS imaging of PGE_2_/PGD_2_ (right) on serial sections of day 6 implantation sites. M, mesometrial pole; AM, anti-mesometrial pole; LE, luminal epithelium; GE, glandular epithelium; S, stroma. Asterisks indicate embryos. Scale bar: 200 μm. Day 6 (1000 hours) indicates 10 am on day 6 of pregnancy. Three independent sections were assessed for immunostaining and MS imaging. (**C**) Coimmunostaining of CK-8 (a marker of trophoblasts and epithelia) and E-cadherin (a marker of epithelia) showing defective embryo invasion in *COX2*-uKO mice on day 6. Asterisks indicate embryos; arrowheads indicate invading trophoblasts. Scale bar: 100 μm. Three independent sections were assessed for each genotype. Day 6 (1000 hours) indicates 10 am on day 6 of pregnancy. (**D**) Representative images of day 6 uteri from control and *COX2-*uKO mice with or without PGE_2_ analog/DP1 agonist administration. Arrowheads indicate faint implantation sites. Scale bar: 5 mm. *n* = 3 per group. Day 6 (1000 hours) indicates 10 am on day 6 of pregnancy. (**E**) 3D images of luminal and glandular epithelia around embryo implantation sites from control and *COX2-*uKO mice with or without PGE_2_ analog/DP1 agonist administration on day 6 of pregnancy. Asterisks indicate embryos. Scale bar: 200 μm. Three independent uteri were assessed in each group. Day 6 (1000 hours) indicates 10 am on day 6 of pregnancy.

In contrast with the evident expression of COX1 before embryo attachment (Figure 1G), unique spatial expression of COX2 as well as PG receptors was found during the invasion phase (Figure 2E), leading us to examine the expression patterns of PGE_2_ and PGD_2_ synthetic enzymes in this milieu (Supplemental Figure 3). PGH_2_, synthesized from AA by COXs, is converted by specific PG synthases into various PG species that exert actions by binding to specific receptors (4, 7). In agreement with the widespread expression of PGE_2_ receptors in the endometrium (Figure 2E), PGE_2_ synthases (*Ptges*, *Ptges2*, and *Ptges3*) were widely detected in day 6 uteri (Supplemental Figure 8). In contrast, we observed local expression of hematopoietic PGD_2_ synthase (*Hpgds*) in the luminal epithelium (Supplemental Figure 8), which agreed well with the DP expression sites near the embryo attachment sites (Figure 2E). LC-MS/MS data also revealed PG expression in day 6 implantation sites, which were compared between control and *COX2*-uKO mice (Figure 5A). PGD_2_ and PGE_2_ levels were markedly reduced in *COX2*-uKO uteri. PGF_2α_ levels were also decreased in *COX2*-uKO uteri, although they were substantially lower than those of PGD_2_ and PGE_2_. These results indicated that, on day 6, uterine PG synthesis depends entirely on COX2, in contrast with on day 4, where uterine PG synthesis largely depends on COX1 (Figure 4A). While we observed high expression levels of *Txba2r* (encoding a TxA_2_ receptor) in the SDZ (Figure 2E), the TxA_2_ metabolite was not detected (Figure 5A). It is possible that TxA_2_ in the circulating blood acts on the TxA_2_ receptor expressed in decidual blood vessels. These data suggested that COX2 is responsible for the massive production of PGE_2_ and PGD_2_ in day 6 uteri and contributes to the actions exerted by these PGs on day 6.

Imaging MS provided profound insights into the localization of PGE_2_ and PGD_2_ in the decidua (Figure 5B). Evident signals of PGE_2_ and PGD_2_ aligned along the luminal layers around the attached embryo, which was consistent with COX2 expression, as indicated by immunostaining (Figure 5B) and spatial transcriptome analysis (Figure 2E). Based on the spatial expression profiles of the specific synthases and receptors for PGE_2_ and PGD_2_ (Figure 2E and Supplemental Figure 8), PGD_2_ appears to function locally, within the vicinity of the embryos, whereas PGE_2_ likely acts after spreading out toward the anti-mesometrial pole of the decidua (Figure 5B). Taken together, these results demonstrated that COX1 and COX2 differentially contribute to PG synthesis in each phase of pregnancy, and specific receptors with unique localizations exist for each PG species.

The findings of the spatial transcriptome and lipidome analyses raised the question of how uterine COX2 influences implantation processes, especially on day 6 of pregnancy (Figure 5C). After embryo attachment, cell-cell adhesion between luminal epithelial cells in the vicinity of the embryo becomes weaker, contributing to subsequent trophoblast invasion into the decidua (13). Embryo invasion was evident on the morning of day 6, when CK-8–positive trophoblasts laterally infiltrated the deciduae (Figure 3C) (13, 26). However, in contrast with control uteri, *COX2*-uKO uteri showed defective epithelial cell removal, accompanied by anomalous trophoblast invasion (Figure 5C), indicating the importance of COX2 in this process. The spatial gene expression analyses and PG synthesis–related gene expression profiling allowed us to further investigate the individual roles of PGE_2_ and PGD_2_ in the uterus during the embryo invasion phase (Figure 5, D and E). To examine the effects of each PG species in COX2-dependent decidual functions, we subcutaneously injected *COX2*-uKO females with a PGE_2_ analog or a DP1 (PGD_2_ receptor) agonist on the morning of day 5. Compared with the control (no injection), both the PGE_2_ analog and the DP1 agonist enhanced blue reactions in the embryo implantation sites (Figure 5D). We also observed 3D structures of the implantation sites in each group, as a flawed formation of implantation chamber is related to failed embryo invasion, owing to defects of morphological changes in uterine epithelia (21, 33). As shown above (Figure 3A), shallow implantation chambers were found in the *COX2*-deleted milieu (Figure 5E). However, this abnormality was recovered upon administration of the PGE_2_ analog or DP1 agonist, resulting in deeply evaginated LE with elongated glands (Figure 5E). These results indicated the critical roles of PGE_2_ and DP1 signaling in the formation of implantation chambers. These results demonstrated the role of the COX2/PGE_2_/PGD_2_ axis in the establishment of appropriate implantation chambers, facilitating embryo invasion of the decidua.

## Discussion

Embryo implantation, which involves intimate reciprocal communication between the endometrium and embryo, is key for pregnancy maintenance and healthy birth (1). Recently, spatial transcriptome analyses of fetomaternal interfaces have been conducted in humans and mice (34–36), providing previously unknown insights into how maternal tissues communicate with embryos in early pregnancy. However, these previous studies utilized endometria with fully invaded trophoblasts; therefore, the spatiotemporal gene landscapes working during the peri-implantation phase remain unclear. In this study, we analyzed mouse uterine tissues immediately before and after embryo attachment, showing that lipid metabolism–related pathways were enriched in LEs and PDZs directly in contact with embryos upon embryo spacing, attachment, and invasion.

It is well recognized that the intake of NSAIDs during early pregnancy compromises embryo implantation, resulting in embryo absorption (4). COX1 and COX2, the targets of NSAIDs, are the rate-limiting enzymes of PG synthesis. COXs utilize AA provided by PLA_2_s to produce PGG_2_, which is immediately converted to PGH_2_ in cells (5–7). PG synthases specific for each PG are responsible for the generation of bioactive PGs from PGH_2_, triggering GPCR signaling in an autocrine or paracrine manner. In vitro studies have demonstrated that PLA_2_s, COXs, and specific PG synthases show preferential coupling (7). Uteri of pregnant animals are remarkable in vivo models to investigate the differential roles of COX1 and COX2. Both enzymes are spatiotemporally expressed in the uterus during the peri-implantation period, with mutually exclusive expression patterns (8, 37). A previous study using reciprocal *COX1*- and *COX2*-knockin mice revealed reduced fertility, with defective embryo implantation (38); the authors prepared mice with knockin alleles of *COX1* at the *COX2* loci (*COX1* > *COX2*) as well as reciprocally knocking in alleles of *COX1* and *COX2* (Reversa). Intriguingly, Reversa females exhibited a more severe phenotype during embryo implantation than *COX1* > *COX2* females, indicating that both COX1 and COX2 contribute to early pregnancy outcomes. These results suggested that each COX enzyme has a unique and specific role in the pregnant uterus.

This study revealed that COX1 is responsible for the synthesis of PGE_2_, PGD_2_, PGI_2_, and TxA_2_ in the receptive endometrium, while COX2 mainly produces PGE_2_ and PGD_2_ during embryo invasion. It remains unclear exactly how each enzyme is related to the synthesis of unique PGs in vivo, but our observations in day 6 uteri indicated that COX2 may be related to hematopoietic PGD_2_ synthase (*Hpgds*). Intriguingly, PGE_2_-producing enzymes did not colocalize with COX2 during the invasion phase. As COX2-produced PGH_2_ is unstable, it has to be immediately converted into each PG by specific synthases. Therefore, the question arises as to how COX2 couples with PGE_2_ synthases in early pregnant uteri. As shown in a recent study (39), it is possible that PGs, including PGH_2_, are transferred to cells expressing specific PG synthases and receptors via extracellular vesicles.

Our current study also demonstrates previously unappreciated roles of COX1 in early pregnancy. Notably, abnormal embryo spacing was found in *COX1*/*COX2*-DKO uteri, but not in *COX2*-uKO uteri, indicating the involvement of COX1-producing PGs in this process. However, which PG species play a major role remains elusive. There are reports showing that muscle contractions contribute to embryo spacing (40, 41), indicating that PGs might induce muscle contraction by being distributed throughout the endometria, while COX1 is expressed in LE. Indeed, we observed reduced production of 6-keto-PGF_1α_ (a metabolite of PGI_2_) and the absence of TxB_2_ (a metabolite of TxA_2_) in *COX1*/*COX2*-DKO uteri on day 4, in addition to myometrial expression of their receptors. In contrast, accumulating studies using 3D imaging have shown that planar polarity in epithelia is another important factor regulating embryo spacing (28, 29). Therefore, we cannot ignore the possibility that PGs synthesized by COX1 work on LE. As single deletion of each PG receptor does not influence uterine functions (4), multiple PG receptors might cooperate to regulate embryo spacing. Another evident phenotype in *COX1*/*COX2*-DKO is flawed embryo attachment accompanied with abolished blue dye reactions. As embryo attachment induces increased vascular permeability in the endometria, embryo-attached sites can be visualized by the injection of blue dye (23). Matsumoto et al. reported that systemic deletion of *COX2* causes reduced angiogenesis in the endometria (42), indicating possible roles of PGs in endothelia or immune cells. As previously reported (43), endometria and endothelia can have opposite effects against pregnancy outcomes, and thus it would be intriguing to see whether endothelial cell–specific deletion of COX1 or COX2 has any roles in early pregnancy.

Interestingly, defective implantation in *COX2*-uKO mice was rescued by a single administration of a DP1 agonist. While PGE_2_ can trigger various G protein signaling pathways, including Gs activation, via 4 PGE receptor subtypes, the DP1 agonist only triggers Gs signaling via the DP1 receptor; thus, PGE_2_ and the DP1 agonist may share Gs signaling activation (4). Our result suggests a critical role for the increased cAMP in decidualization; indeed, both *Ptger2* and *Ptgdr* were expressed in luminal epithelial cells in day 6 uteri (Figure 5B). A previous study reported that the the COX2/PGE_2_ axis contributes to decidual reactions by upregulating stromal cAMP levels downstream of EP2 and EP4 (44). In addition, cAMP is required in primary culture of human uterine stroma to support decidualization in vitro (45). These findings suggest that PGE_2_ and PGD_2_ may exert their functions mainly via Gs-coupled receptors. This would also explain how COX2/PG signaling elicited a transformation in epithelial morphology (Figure 5, D and E). Recently, Ishihara et al. reported that PGE_2_/EP2/cAMP signaling is involved in YAP-induced cell competition in epithelial Madin-Darby canine kidney cells. In epithelial cells in which YAP was activated by physical stimuli, COX2 was induced to produce PGE_2_, which induced E-cadherin internalization only in PG-producing cells and adjacent cells in an EP2/cAMP-dependent manner, thereby eliminating YAP-activated cells (46). As EP2 and DP1 receptors share Gs activation, it is possible that uterine epithelia transform their shapes via the PGE_2_/PGD_2_/EP2/EP4/DP1/cAMP axis. As a previous study showed prominent localization of YAP in LE as well as PDZ cells, which exhibit epithelial cell–like features (21), such a system may also work in the endometrium during early pregnancy. Exogenous administration of PGE_2_ or PGD_2_ helped recover decidual reactions, but failed to rescue embryo growth in *COX2*-uKO mice. If a cell-competitive mechanism occurs sequentially and away from the embryo attachment site during epithelial breakdown, it requires the formation of a local PG concentration gradient, which may be difficult to achieve with exogenous PG administration. Mouse models with spatiotemporal knockout or induction of PG-related enzymes would be useful to further investigate this in the future.

In conclusion, the current study showed that lipid metabolism pathways, especially 2 COX isoforms (COX1 and COX2), differentially contribute to peri-implantation events in the mouse uterus in a spatiotemporal manner. This study revealed that PG species perform unique functions downstream of COX1 and COX2 to facilitate embryo spacing, attachment, and invasion. As COX1 and COX2 are also found in the human endometrium during implantation (47), the mechanism found in mice may be conserved among species, including humans.

## Methods

### Sex as a biological variable.

We utilized female mice for our analyses, as pregnancy occurs only in females.

### Mice.

*Ptgs2* (*COX2*)*-loxP/loxP*, *Pgr-Cre*, and *Ptgs1* (*COX1*)-KO mice were used in this study. *Ptgs2^fl/fl^* mice (48) were provided by Harvey R. Herschman (UCLA, Los Angeles, California, USA). *Ptgs1*-KO mice (49) were supplied by Taconic Biosciences. *Pgr-Cre* mice (50) were provided by Francesco J. DeMayo (National Institute of Environmental Health Sciences, Research Triangle Park, North Carolina, USA) and John P. Lydon (Baylor College of Medicine, Houston, Texas, USA). *Ptgs2-loxP/loxP* females were crossed with *Pgr-Cre* males to generate *COX2*-uKO mice. *COX2*-uKO males were further mated with *COX1*-KO females to establish *COX1*/*COX2*-DKO mice. All mice were housed under a 12-hour light/12-hour dark cycle with lights on from 08:00 to 20:00 hours in humidity-controlled rooms at 22°C at the University of Tokyo Animal Care Facility according to the institutional guidelines for the use of laboratory animals.

### Evaluation of pregnancy outcomes.

To examine pregnancy outcomes, *COX2*-uKO, *COX1*/*COX2*-DKO, or *COX2^fl/fl^* (control) female mice were mated with C57BL/6N fertile male mice, as reported in a previous study (12, 30, 51). The day when the vaginal plug was detected was considered as day 1 of pregnancy. Pregnant mice were euthanized by cervical dislocation on designated days of pregnancy for the evaluation of pregnancy phenotypes and sample collection. On day 4, one uterine horn was flushed with saline to confirm the presence of blastocysts. Embryo attachment sites were observed as blue bands soon after intravenous injection of 1% solution of Chicago blue dye (Sigma-Aldrich) in saline on days 5 and 6. When no embryo attachment sites were observed as of day 5, both uterine horns were cut and flushed with saline to collect embryos.

### DNA/RNA extraction.

DNA and RNA were extracted from homogenized tissues using TRI Reagent (Molecular Research Center Inc.) according to the manufacturer’s protocol. The concentrations of each sample were tested using a NanoDrop spectrophotometer (Thermo Fisher Scientific). The complementary DNA was synthesized from the extracted RNA using ReverTra Ace qPCR RT Master Mix with gDNA Remover (TOYOBO).

### PCR to check genomic deletion of COX2.

DNA collected from uterine tissues was used as a template. One hundred nanograms of DNA was amplified using the primers 5′-AATTACTGCTGAAGCCCACC-3′ and 5′-GAATCTCCTAGAACTGACTGG-3′ and rTaq (Takara), with 35 cycles of 98°C for 30 seconds, 58°C for 30 seconds, and 72°C for 30 seconds. The PCR products were electrophoresed in a 2% agarose gel to detect a band shift.

### Quantitative reverse transcription PCR.

qPCRs were run using THUNDERBIRD SYBR qPCR Mix (TOYOBO). The housekeeping gene *Actb* was used for internal standardization of mRNA expression. Relative expression levels were determined by the ΔΔCt method (52). The following primers were used: *Ptgs1* 5′-TCCAGGAGCTCACAGGAGA-3′ and 5′-GTCACCACCGAACGTGCT-3′; *Hdc* 5′-GCCCATCTGTGCCAGTGAGGGA-3′ and 5′-GAAAGCGCCGGCTCAAGGGG-3′; *Ihh* 5′-GAGAACACGGGTGCCGACCG-3′ and 5′-CAGCGGCCGAATGCTCAGACT-3′; *Muc1* 5′-GTGCCAGTGCCGCCGAAAGA-3′ and 5′-CCGCCAAAGCTGCCCCAAGT-3′; *Actb* 5′-TGTTACCAACTGGGACGACA-3′ and 5′-GGGGTGTTGAAGGTCTCAAA-3′.

### Immunofluorescence.

Frozen sections (thickness, 12 μm) were prepared by CM1950 cryostat (Leica) and used for immunofluorescence. After fixation in 4% paraformaldehyde in PBS, the sections were incubated with primary antibodies against Ki67 (SP6, Thermo Fisher Scientific; 1:300), COX2 (160106, Cayman; 1:300), E-cadherin (24E10, Cell Signaling Technology; 1:300), and CK-8 (TROMA-I, DSHB; 1:300). Signals were detected using Alexa Fluor 555–conjugated anti–rabbit IgG (A21428, Thermo Fisher Scientific; 1:500), Alexa Fluor 488–conjugated anti–rat IgG (A11006, Thermo Fisher Scientific; 1:500), and 4′,6-diamidino-2-phenylindole (DAPI) (Dojindo, 1:500). Images were acquired using an AXR confocal microscopy system (Nikon).

### FISH.

We performed FISH as previously reported (21, 53), with some modifications. Briefly, frozen sections with 12 μm thickness were attached on Superfrost Plus Microscope Slides (Thermo Fisher Scientific). After fixation and acetylation, the sections were hybridized overnight at 55°C with a DIG-labeled *Bmp2* antisense probe. Following probe washing, the sections were treated with an anti-DIG antibody-POD (11207733910, Roche; 1:200), followed by Alexa Fluor 488–conjugated tyramide (B40953, Invitrogen). Nuclei were counterstained with DAPI. Images were acquired using an AXR confocal microscopy system.

### LC-MS/MS.

Pregnant uteri were collected in the morning of days 4 and 6 and processed for LC-MS/MS–based lipidome analyses, as previously described (54). Briefly, collected tissues were snap-frozen and homogenized in methanol, and lipids were extracted by single-phase extraction. Lipidome analysis was performed using the ACQUITY UPLC system (Waters) coupled with quadruple time-of-flight/MS (TripleTOF 5600 or 6600, SCIEX).

### Imaging MS analysis.

Fresh-frozen sections (12 μm thick) of day 6 implantation sites were attached to a conductive indium tin oxide–coated glass slide (Matsunami Glass Industries). For on-tissue derivatization prior to matrix coating, Girard T reagent (G900-100G, Merck), which converts the ketone groups of PGE_2_ and PGD_2_ into the positively charged hydrazone, was prepared at 10 mg/mL in 20% acetic acid (Fujifilm Wako Pure Chemical Corporation). One milliliter of Girard T reagent was sprayed onto each glass slide using an artist’s airbrush (PS-270, GSI Creos). The sections were then airbrushed with DHB matrix coating (490-79-9, Bruker, 50 mg/mL in 80% ethanol). Imaging MS with ion mobility separation was performed using a matrix-assisted laser desorption/ionization-time-of-flight instrument (timsTOF flex, Bruker Daltonics). The tissue surface was irradiated with a YAG laser (500 laser shots/pixel) in the positive ion detection mode and the spot-to-spot center distance was set to 35 μm. The ion mobility conditions were optimized to separate the ions of Girard T–derivatized PGE_2_ and PGD_2_ by measuring reference standards. The obtained MS spectra were reconstructed into MS images using the Scils Lab software (Bruker Daltonics).

### Spatial transcriptome analysis.

Spatial transcriptome analysis was performed using 10× Visium (10× Genomics) according to the manufacturer’s protocol. Briefly, frozen sections (10 μm thick) were mounted on gene expression slides and sent to KOTAI Biotechnologies for subsequent procedures. After reaction with Proteinase K for 40 minutes, the sections were hybridized with spatial tags on the slides and reverse transcribed in situ. The synthesized cDNAs were subjected to RNA-seq using DNBseq (MGI), producing 300 million reads/sample.

Raw FASTQ files and microscope slide images for each sample were processed using the Space Ranger software (version 1.1, 10× Genomics) with the spaceranger count pipeline, using STAR with default parameters for aligning reads against the mouse reference genome mm10 refdata-gex-mm10-2020-A. This pipeline uses the Visium spatial barcodes to generate a feature-spot matrix of unique molecular identifier counts. To visualize genes of interest, we used Loupe Browser (10× Genomics) according to the manufacturer’s protocol. GO enrichment was analyzed by Metascape with the default setting (https://metascape.org/) (55).

### Analysis of published scRNA-seq data.

To validate our cell clustering data obtained with 10× Visium, we analyzed previously published scRNA-seq data sets (15) for day 4 mouse uteri (3.5 dpc in the original paper). Raw single-end RNA-seq reads were aligned to the indexed mouse genome (GRCm38/mm10) using DropSeqTools (https://github.com/broadinstitute/Drop-seq) with default settings. Reanalyses of scRNA-seq data were conducted using the Seurat package for R (v.5.1.0) (56).

### 3D visualization of implantation sites.

3D visualization of day 6 implantation sites was performed as previously reported (29). To stain luminal and glandular epithelial cells, day 6 tissues were incubated with anti–E-cadherin antibodies (24E10, Cell Signaling Technology; 1:500) followed by incubation with an anti-rabbit antibody conjugated with Alexa Fluor 555 (A21428, Thermo Fisher Scientific; 1:500). 3D images were acquired using LSM 880 (Zeiss) and AXR (Nikon) microscopes. To construct a 3D structure from the images, the surface tool in Imaris (version 9.8, Oxford Instruments) was used.

### Measurement of serum P_4_ levels.

Blood samples were collected from mice on the indicated days of pregnancy. Serum P_4_ levels were measured as described previously (26), using a progesterone EIA kit (582601, Cayman).

### Statistics.

Statistical analyses were performed using a 2-tailed Student’s *t* test or 1-way ANOVA followed by Bonferroni’s post hoc test in GraphPad Prism 10. Statistical significance was set at a *P* value of less than 0.05.

### Study approval.

All animal experiments were approved by the Institutional Animal Experiment Committee of the University of Tokyo Graduate School of Medicine (approval numbers P16-066, P20-076, and A2023M165).

### Data availability.

RNA-seq data reported in this study were deposited to the NCBI Gene Expression Omnibus (GEO accession no. GSE253520). Values for all data points in the graphs are provided in the supplemental Supporting Data Values file.

## Author contributions

S Aikawa, MM, S Akaeda, and YH designed the study. S Aikawa, MM, S Akaeda, MA, YI, Y Sugiura, ST, RM, DH, XH, CI, RI, YF, and TH performed the experiments and collected the data. S Aikawa, MM, S Akaeda, MA, YI, Y Sugiura, and YH analyzed the data. Y Sugimoto, RSH, NT, MH, OWH, and YO discussed and interpreted the results. S Aikawa drafted the manuscript, which was edited by YH and Y Sugimoto. RSH critically reviewed the manuscript. YH supervised the study.

## Supplementary Material

Supplemental data

Supplemental table 1

Supplemental table 2

Supplemental table 3

Supplemental table 4

Supporting data values

## Version 1 10/08/2024

Electronic publication

## References

[1] Cha J (2012). Mechanisms of implantation: strategies for successful pregnancy. Nat Med.

[2] Fukui Y (2019). Uterine receptivity, embryo attachment, and embryo invasion: multistep processes in embryo implantation. Reprod Med Biol.

[3] Hama K (2007). Embryo spacing and implantation timing are differentially regulated by LPA3-mediated lysophosphatidic acid signaling in mice. Biol Reprod.

[4] Sugimoto Y (2015). Roles of prostaglandin receptors in female reproduction. J Biochem.

[5] Dubois RN (1998). Cyclooxygenase in biology and disease. FASEB J.

[6] Aikawa S, Hirota Y (2024). Roles of lipid mediators in early pregnancy events. Reprod Med Biol.

[7] Murakami M, Kudo I (2004). Recent advances in molecular biology and physiology of the prostaglandin E2-biosynthetic pathway. Prog Lipid Res.

[8] Song H (2002). Cytosolic phospholipase A2alpha is crucial [correction of A2alpha deficiency is crucial] for ‘on-time’ embryo implantation that directs subsequent development. Development.

[9] Reese J (2000). Coordinated regulation of fetal and maternal prostaglandins directs successful birth and postnatal adaptation in the mouse. Proc Natl Acad Sci U S A.

[10] Gross GA (1998). Opposing actions of prostaglandins and oxytocin determine the onset of murine labor. Proc Natl Acad Sci U S A.

[11] Lim H (1997). Multiple female reproductive failures in cyclooxygenase 2-deficient mice. Cell.

[12] Akaeda S (2021). Retinoblastoma protein promotes uterine epithelial cell cycle arrest and necroptosis for embryo invasion. EMBO Rep.

[13] Li Y (2015). Entosis allows timely elimination of the luminal epithelial barrier for embryo implantation. Cell Rep.

[14] Hirota Y (2019). Progesterone governs endometrial proliferation-differentiation switching and blastocyst implantation. Endocr J.

[15] Wang HQ (2023). Maternal and embryonic signals cause functional differentiation of luminal epithelial cells and receptivity establishment. Dev Cell.

[16] Hirai H (2001). Prostaglandin D2 selectively induces chemotaxis in T helper type 2 cells, eosinophils, and basophils via seven-transmembrane receptor CRTH2. J Exp Med.

[17] Forman BM (1997). Hypolipidemic drugs, polyunsaturated fatty acids, and eicosanoids are ligands for peroxisome proliferator-activated receptors alpha and delta. Proc Natl Acad Sci U S A.

[18] Lim H, Dey SK (2000). PPAR delta functions as a prostacyclin receptor in blastocyst implantation. Trends Endocrinol Metab.

[19] Hara S (2017). Prostaglandin terminal synthases as novel therapeutic targets. Proc Jpn Acad Ser B Phys Biol Sci.

[20] Paria BC (1999). Zonula occludens-1 and E-cadherin are coordinately expressed in the mouse uterus with the initiation of implantation and decidualization. Dev Biol.

[21] Yuan J (2019). Primary decidual zone formation requires Scribble for pregnancy success in mice. Nat Commun.

[22] Goolam M (2020). The transcriptional repressor Blimp1/PRDM1 regulates the maternal decidual response in mice. Nat Commun.

[24] Hirota Y (2010). Uterine-specific p53 deficiency confers premature uterine senescence and promotes preterm birth in mice. J Clin Invest.

[25] Fukui Y (2023). The EZH2-PRC2-H3K27me3 axis governs the endometrial cell cycle and differentiation for blastocyst invasion. Cell Death Dis.

[26] Matsumoto L (2018). HIF2α in the uterine stroma permits embryo invasion and luminal epithelium detachment. J Clin Invest.

[27] Aikawa S (2019). Uterine deficiency of high-mobility group box-1 (HMGB1) protein causes implantation defects and adverse pregnancy outcomes. Cell Death Differ.

[28] Arora R (2016). Insights from imaging the implanting embryo and the uterine environment in three dimensions. Development.

[29] Yuan J (2018). Tridimensional visualization reveals direct communication between the embryo and glands critical for implantation. Nat Commun.

[30] Fukui Y (2021). Uterine epithelial LIF receptors contribute to implantation chamber formation in blastocyst attachment. Endocrinology.

[31] Flores D (2020). Mechanical and signaling mechanisms that guide pre-implantation embryo movement. Development.

[32] Ye X (2005). LPA3-mediated lysophosphatidic acid signalling in embryo implantation and spacing. Nature.

[33] Li Y (2019). Mice missing Cnr1 and Cnr2 show implantation defects. Endocrinology.

[34] Greenbaum S (2023). A spatially resolved timeline of the human maternal-fetal interface. Nature.

[35] Arutyunyan A (2023). Spatial multiomics map of trophoblast development in early pregnancy. Nature.

[36] Li R (2022). Spatial transcriptomic profiles of mouse uterine microenvironments at pregnancy day 7.5†. Biol Reprod.

[37] Chakraborty I (1996). Developmental expression of the cyclo-oxygenase-1 and cyclo-oxygenase-2 genes in the peri-implantation mouse uterus and their differential regulation by the blastocyst and ovarian steroids. J Mol Endocrinol.

[38] Li X (2018). Isoform-specific compensation of cyclooxygenase (Ptgs) genes during implantation and late-stage pregnancy. Sci Rep.

[39] Kudo K (2022). Secreted phospholipase A_2_ modifies extracellular vesicles and accelerates B cell lymphoma. Cell Metab.

[40] Chen Q (2011). Transient {beta}2-adrenoceptor activation confers pregnancy loss by disrupting embryo spacing at implantation. J Biol Chem.

[41] Dawson M (2024). Imaging the dynamics of murine uterine contractions in early pregnancy†. Biol Reprod.

[42] Matsumoto H (2002). Cyclooxygenase-2 differentially directs uterine angiogenesis during implantation in mice. J Biol Chem.

[43] Deng W (2019). Endothelial cells in the decidual bed are potential therapeutic targets for preterm birth prevention. Cell Rep.

[44] Ruan YC (2012). Activation of the epithelial Na^+^ channel triggers prostaglandin E_2_ release and production required for embryo implantation. Nat Med.

[45] Vinketova K (2016). Human decidual stromal cells as a component of the implantation niche and a modulator of maternal immunity. J Pregnancy.

[46] Ishihara E (2020). Prostaglandin E_2_ and its receptor EP2 trigger signaling that contributes to YAP-mediated cell competition. Genes Cells.

[47] Marions L, Danielsson KG (1999). Expression of cyclo-oxygenase in human endometrium during the implantation period. Mol Hum Reprod.

[48] Ishikawa TO, Herschman HR (2006). Conditional knockout mouse for tissue-specific disruption of the cyclooxygenase-2 (Cox-2) gene. Genesis.

[49] Langenbach R (1995). Prostaglandin synthase 1 gene disruption in mice reduces arachidonic acid-induced inflammation and indomethacin-induced gastric ulceration. Cell.

[50] Soyal SM (2005). Cre-mediated recombination in cell lineages that express the progesterone receptor. Genesis.

[51] Matsumoto H (2016). Molecular and cellular events involved in the completion of blastocyst implantation. Reprod Med Biol.

[52] Livak KJ, Schmittgen TD (2001). Analysis of relative gene expression data using real-time quantitative PCR and the 2(-Delta Delta C(T)) Method. Methods.

[53] Dewar A (2023). Tissue-specific RNA localization in the uterus during implantation, decidualization and placentation: technical nuances of various labeling approaches. Curr Protoc.

[54] Aikawa S (2017). Autotaxin-lysophosphatidic acid-LPA_3_ signaling at the embryo-epithelial boundary controls decidualization pathways. EMBO J.

[55] Zhou Y (2019). Metascape provides a biologist-oriented resource for the analysis of systems-level datasets. Nat Commun.

[56] Stuart T (2019). Comprehensive integration of single-cell data. Cell.

