# Supplementary material for: Spatiotemporally distinct roles of cyclooxygenase-1 and cyclooxygenase-2 at fetomaternal interface in mice
Source: JCI Insight. 2024 Aug 27;9(19):e181865. doi: 10.1172/jci.insight.181865 (PMC11466189; doi:10.1172/jci.insight.181865)
Supplement: Supplemental data [file jciinsight-9-181865-s083.pdf]

# SUPPLEMENTAL MATERIALS

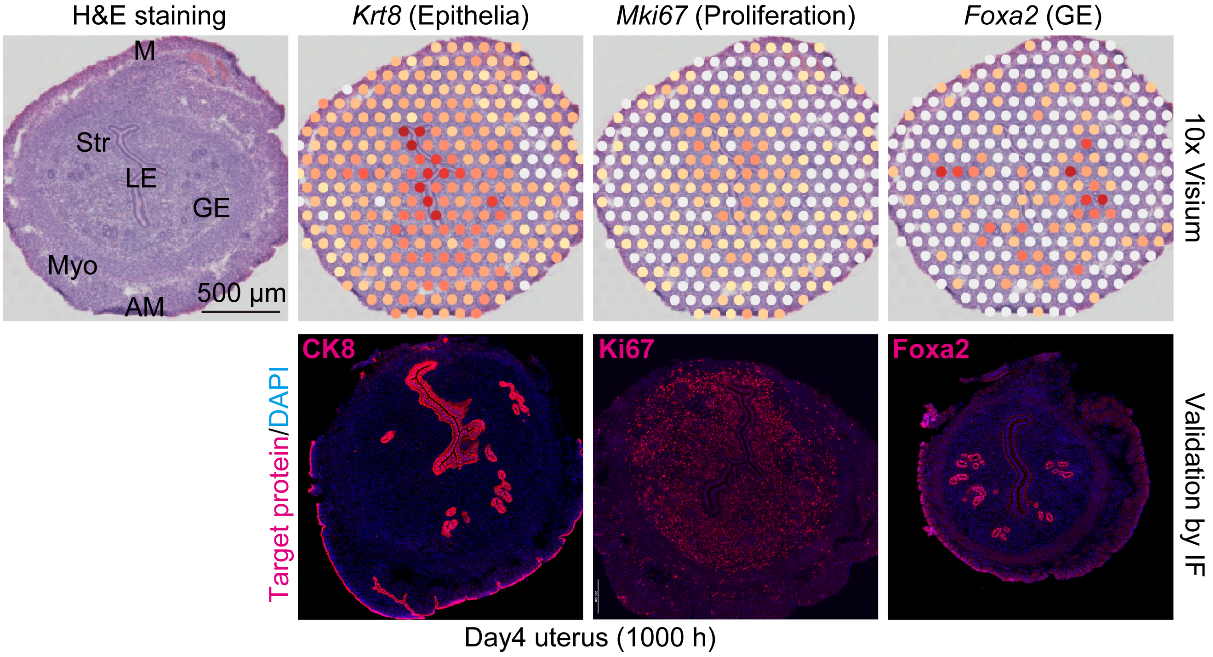


**Supplemental Figure 1. Validation of 10x Visium analysis on day 4 of pregnancy.**

10x Visium data were validated via immunofluorescence of CK8 (*Krt8*; an epithelial marker), Ki67 (*Mki67*; a cell proliferation marker that should be stained in the stroma on day 4), and Foxa2 (a GE marker). M: mesometrium, AM: anti-mesometrium, LE: luminal epithelium, GE: glandular epithelium, Str: stroma, Scale bar: 500 µm. Three independent sections were assessed for each immunofluorescence.

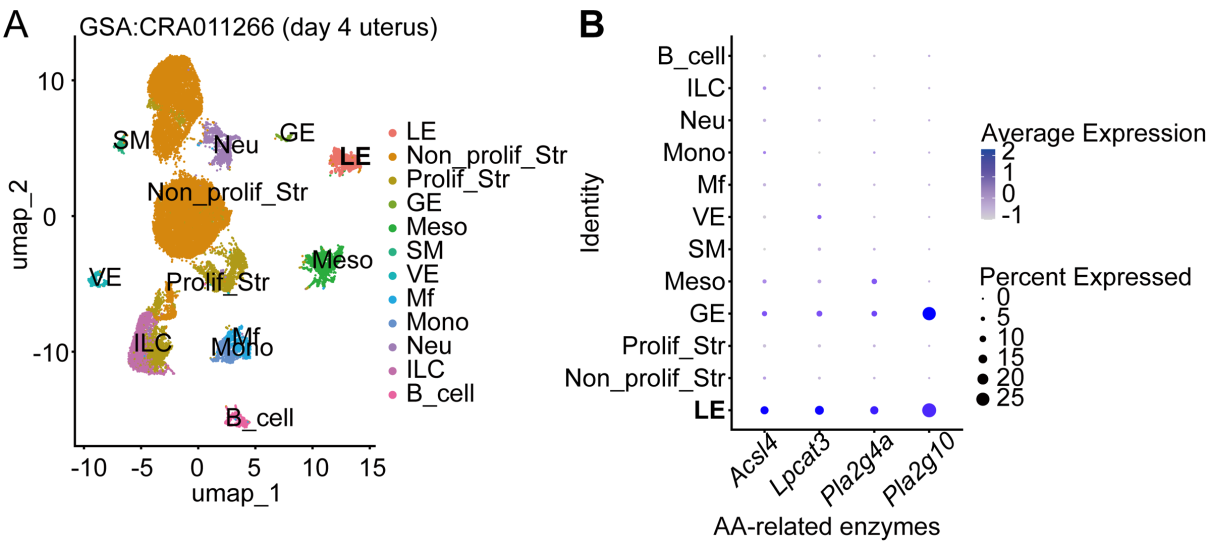


**Supplemental Figure 2. Concentrated expression of arachidonic acid (AA)-related genes in the day 4 LE cluster, as revealed by single-cell RNA-seq.**

**(A)** UMAP projections of day 4 mouse uteri.

**(B)** Dot plot showing the log2 expression of AA-related genes characteristic of each cluster. LE: luminal epithelia, Non_prolif_Str: non-proliferative stroma, Prolif-Str: proliferative stroma, GE: glandular epithelia, Meso: mesothelia, SM: smooth muscle, VE: vascular endothelia, Mf: macrophage, Mono: monocytes, Neu: neutrophil, ILC: innate lymphoid cell, B_cell: B cell.

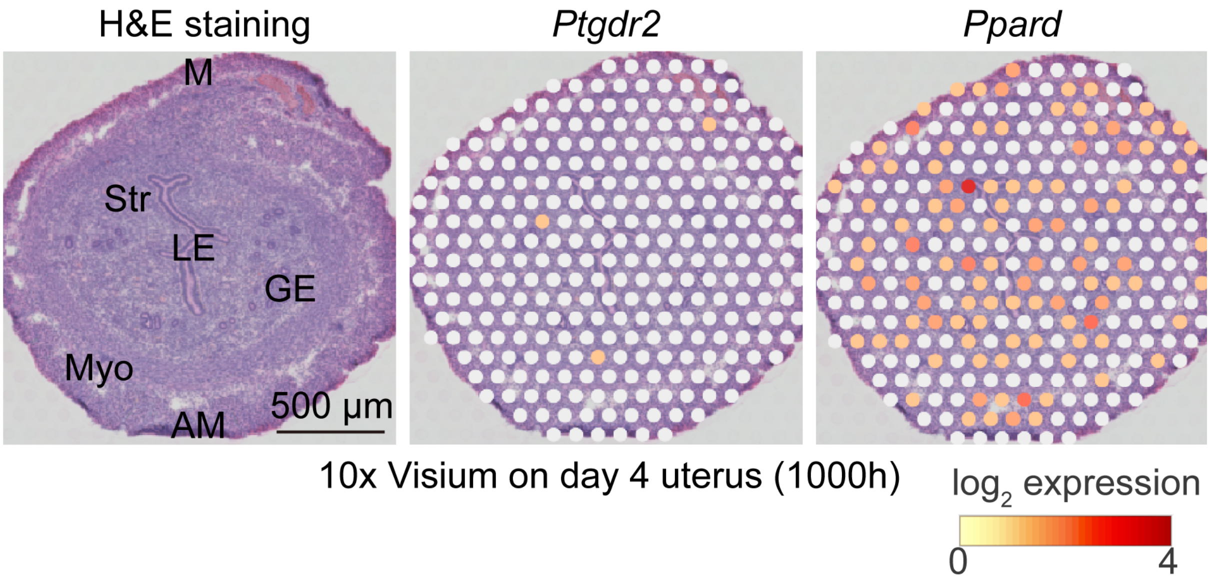


**Supplemental Figure 3. Expression of *Ptgdr2* (the second $PGD_2$ receptor) and Ppard (a possible $PGI_2$ receptor) in day 4 uterus.**

M: mesometrium, AM: anti-mesometrium, LE: luminal epithelium, GE: glandular epithelium, Str: stroma, Scale bar: 500 µm.

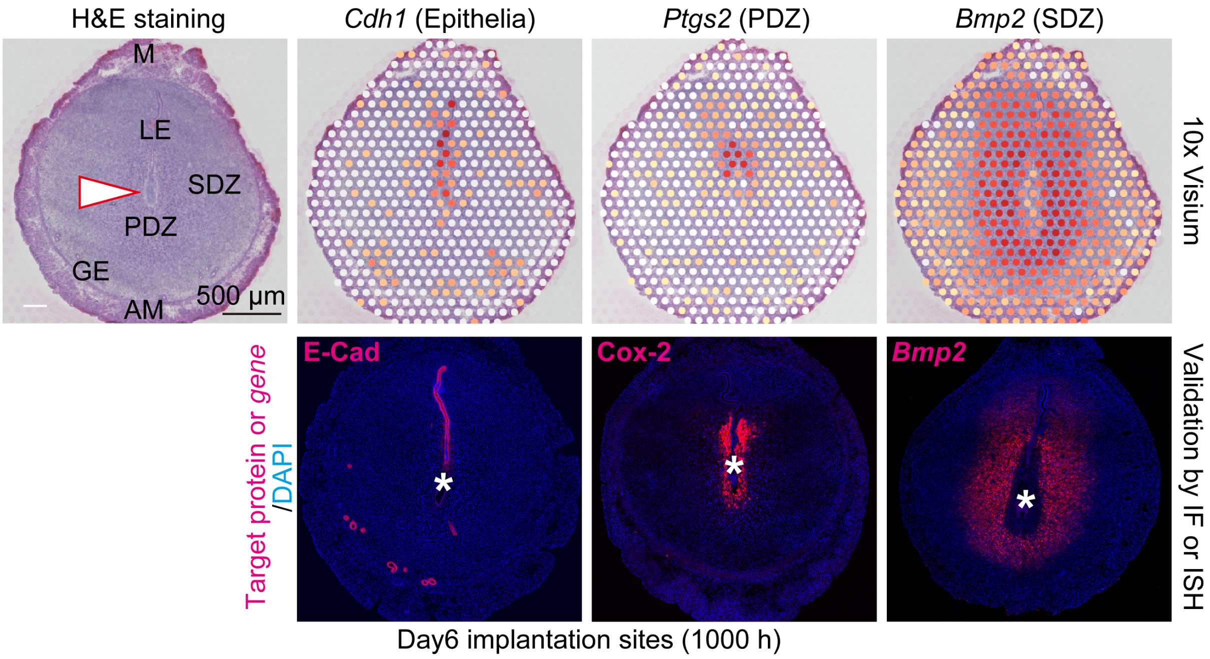


**Supplemental Figure 4. Validation of 10x Visium analysis on day 6 of pregnancy.**

10x Visium data were validated by either immunofluorescence of E-Cad (*Cdh1*; an epithelial marker) and COX2 (*Ptgs2*; a PDZ gene) or *in situ* hybridization (ISH) of *Bmp2* (an SDZ gene). M: mesometrium, AM: anti-mesometrium, LE: luminal epithelium, GE: glandular epithelium, PDZ: primary decidual zone, SDZ: secondary decidual zone. The arrowhead and asterisks indicate embryos. Scale bar: 500 µm. Three independent sections were assessed for each immunofluorescence or ISH.

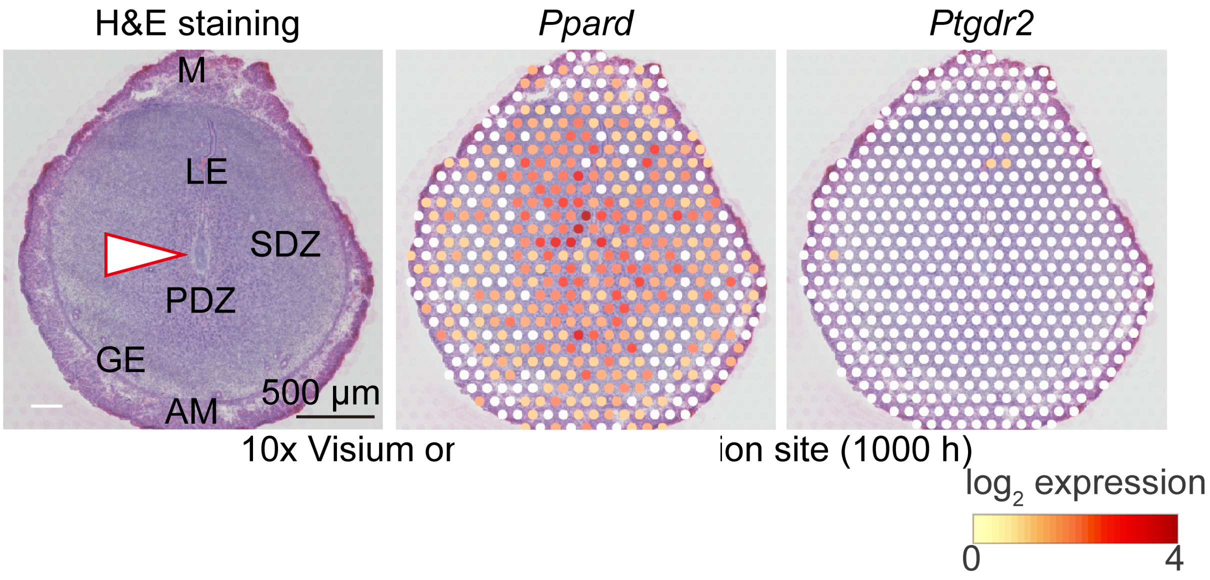


**Supplemental Figure 5. Expression of *Ptgdr2* (the second PGD$_2$ receptor) and Ppard (a possible PGI$_2$ receptor) in day 6 implantation site.**

M: mesometrium, AM: anti-mesometrium, LE: luminal epithelium, GE: glandular epithelium, PDZ: primary decidual zone, SDZ: secondary decidual zone. The arrowhead indicates the attached embryo. Scale bar: 500 µm.

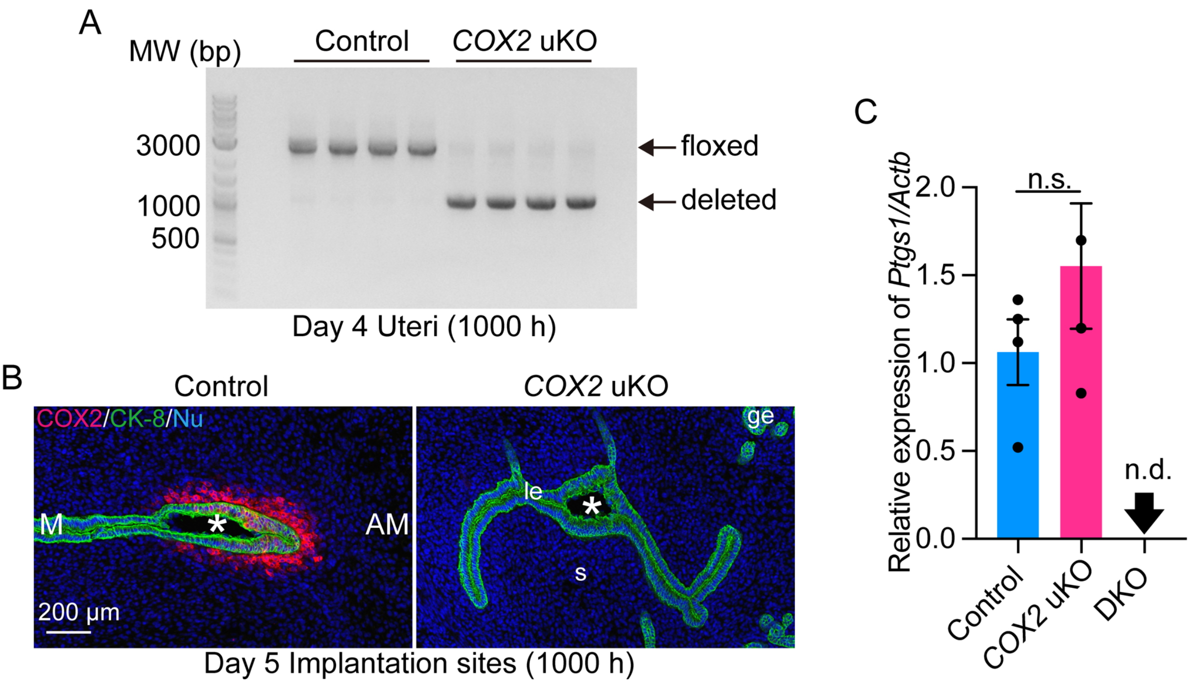


**Supplemental Figure 6. Efficient deletion of COX1 and COX2 in uterine tissues.**

**(A)(B)** *COX2* deletion in the uterus was confirmed via RT-qPCR on day 4 (**A**) and immunostaining on day 5 (**B**). In (**B**), epithelial cells were co-stained with an anti-CK-8 antibody. M: mesometrium, AM: anti-mesometrium, LE: luminal epithelium, GE: glandular epithelium, S: stroma, *: embryo. Scale bar: 200 µm. Three independent sections were assessed for each genotype in (**B**).

**(C)** RT-qPCR confirmed *COX1* deletion (*Ptgs1*) in DKO uteri on day 4 of pregnancy. n = 4 per genotype. Mean ± SEM, n.d.: not detected. n.s.: not significant (Student's *t*-test).

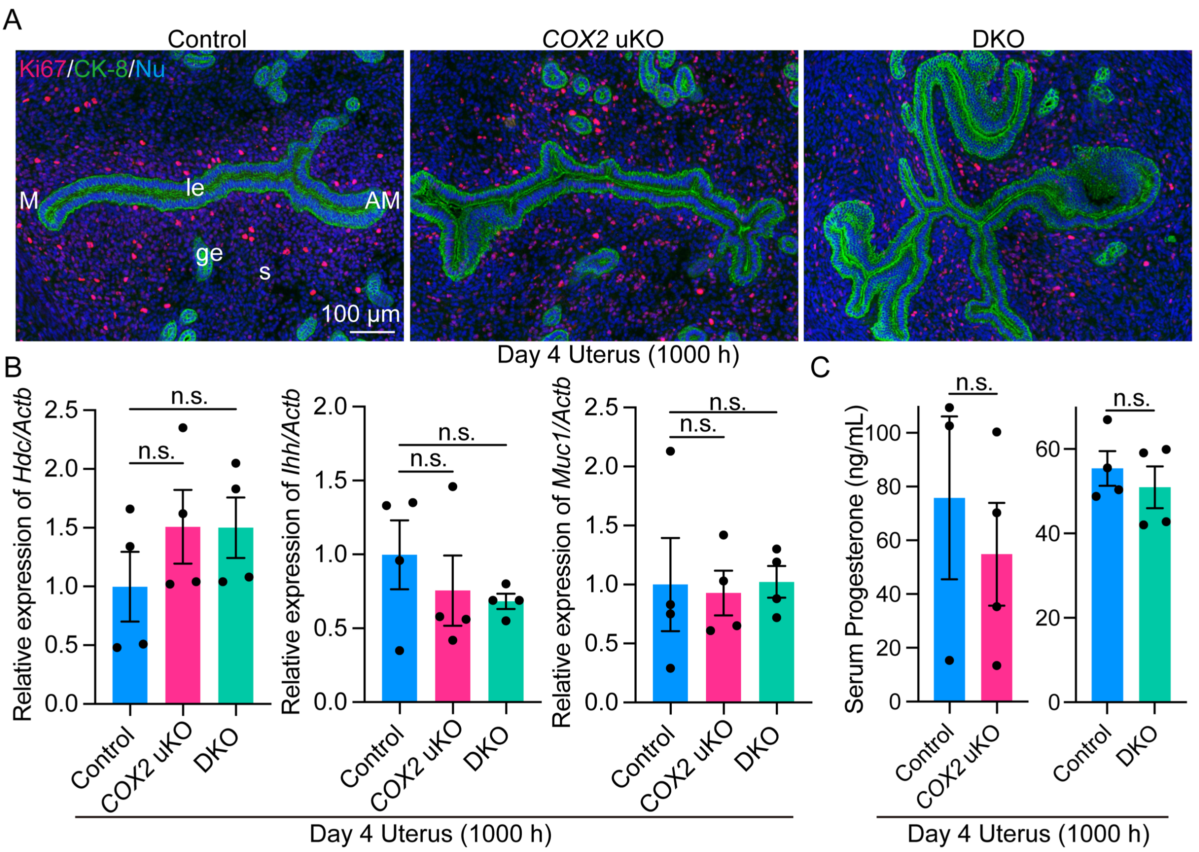


**Supplemental Figure 7. *COX1*/*COX2* DKO does not impair uterine receptivity and ovarian function.**

**(A)** Representative images of the immunostaining of Ki67 (a proliferation marker) and CK-8 showing comparable PDS in each genotype on day 4 of pregnancy. M: mesometrium, AM: anti-mesometrium, LE: luminal epithelium, GE: glandular epithelium, S: stroma. Scale bar: 100 µm. Three independent sections were assessed for each genotype.

**(B)** RT-qPCR revealed comparable levels of the receptive markers *Hdc* (left), *Ihh* (middle), and *Muc1* (right) in day 4 uteri from each genotype. n = 3 per genotype. Mean ± SEM, n.s.: not significant (one-way ANOVA).

**(C)** Serum $P_4$ concentrations on day 4 were not affected by *COX2* uKO (left) or *COX1*/*COX2* DKO (right). n = 3 per genotype. Mean ± SEM, n.s.: not significant (Student's *t*-test).

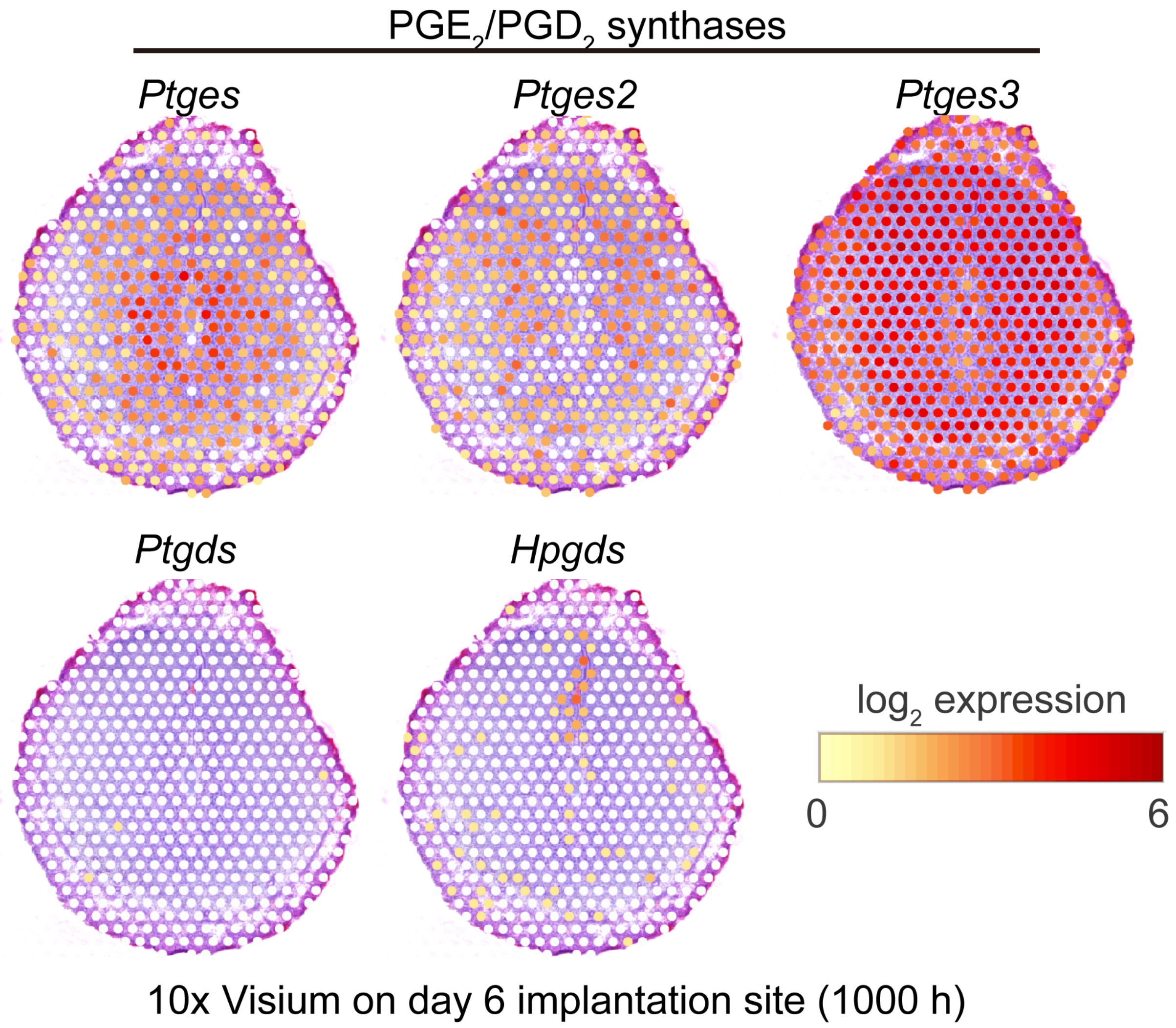


**Supplemental Figure 8. Spatial expression of $PGE_2$/$PGD_2$-specific synthesis-related enzymes during the invasion phase.**

Spatial transcriptomics of day 6 implantation sites showing expression patterns of specific $PGE_2$ and $PGD_2$ synthases. PDZ is encircled by a dashed line.

**Supplemental Table 1. Differentially expressed genes in each cell cluster in day 4 uteri, as revealed by 10× Visium transcriptomics analysis.**

**Supplemental Table 2. Summary of GO enrichment analysis of LE-specific genes on day 4, as revealed by Metascape.**

**Supplemental Table 3. Differentially expressed genes in each cell cluster in day 4 uteri, as revealed by 10× Visium transcriptomics analysis.**

**Supplemental Table 4. Summary of GO enrichment analysis of PDZ-specific genes, as revealed by Metascape.**
